# Spinal cord stimulator explant caused by post-incisional cellulitis secondary to Varicella Zoster Virus (shingles) infection: a case report

**DOI:** 10.1186/s13256-023-04205-4

**Published:** 2023-11-07

**Authors:** Vahid Mohabbati, Mohammadkazem Papan

**Affiliations:** 1 Sydney Pain Research Centre, Suite 213-215 Parkway SAN Clinic, 172 Fox Valley Rd Wahroonga, Sydney, NSW 2076 Australia; 2 Sydney Pain Management Centre, Sydney, NSW Australia

**Keywords:** Spinal cord stimulation (SCS), Surgical site infection (SSI), Varicella Zoster Virus (VZV), Implantable pulse generator (IPG), Case report

## Abstract

**Introduction:**

Spinal Cord Stimulation (SCS) is a well-established therapy for refractory neuropathic pain, known for its safety and minimally-invasive nature. However, complications, including surgical site infections (SSIs), can arise post-implantation. SCS-related SSIs occur in 3.4% to 4.6% of cases within 90 days post-implant, often requiring device removal and impacting pain management and healthcare costs. The impulse generator, electrode implant site and lumbar/thoracic surgical site are commonly affected, with local skin flora and circulating organisms being the primary causes of infection.

**Case presentation:**

An 80-year-old Lebanese male with chronic neuropathic lower back and bilateral leg pain, significantly impairing function, underwent prolonged hospitalizations for COVID-19 infection and acute-on-chronic pain with Urinary Tract Infection (UTI). Considering SCS as a therapeutic option, a successful trial led to permanent implantation, resulting in improved pain severity and functional capacity. However, three months later, the patient developed post-incisional cellulitis and wound dehiscence secondary to Varicella Zoster Virus (shingles) Infection directly over the Implantable Pulse Generator (IPG) incision line. Despite antibiotic treatment, the infection progressed, necessitating SCS system explantation.

**Discussion:**

This represents the first reported case of VZV infection causing wound dehiscence and SCS explantation post-implantation. Contributing factors may include itching around the IPG site, facilitating deeper tissue inoculation. Laboratory and imaging tests may not reliably detect SSIs, and superficial infections may respond to antibiotics, while deep infections typically require implant removal. Early identification and intervention are vital to minimize complications.

**Conclusion:**

This unique case emphasizes the need for heightened vigilance and monitoring in patients with viral infections near medical devices. A standardized approach to assessing and managing SCS-related infections is critical. Sharing such experiences contributes to improved understanding and treatment of these rare incidents.

## Introduction

Spinal Cord Stimulation (SCS) is a well-recognized therapy for the management of refractory neuropathic pain and is generally regarded as a safe and minimally-invasive therapy [1]. As with any surgical procedure, there are known potential complications and risks. Surgical site infections (SSIs) SCS SSI rates ranged from 3.4 to 4.6%, with the majority of cases occurring within 90 days post-implant [2].

In recent years, there has been a growing body of literature on infections related to spinal cord stimulation (SCS). While most literature focuses on bacterial infections, some studies also discuss viral infections. One study reported a fulminant central nervous system (CNS) VZV infection in an HIV-infected patient without the typical VZV-associated rash. The diagnosis of CNS VZV infection was unexpectedly identified. This case highlights the challenges in diagnosing VZV infections when typical skin manifestations are absent [3]. Regarding SCS-related infections, another study reported a rare cervical epidural abscess due to spinal cord stimulation lead implantation. The study highlighted that spinal epidural abscess is rare, with only two cases documented in the literature [4].

Often, SCS infection requires the removal of the device, preventing patients from receiving adequate pain management and adding to the initial health costs and patient disability [2]. An impulse generator (IPG), electrode implant site and lumbar or thoracic surgical site are amongst the most commonly infected areas. Most deep infections occur at the internal pulse generator implantation site (54%), followed by the lead implantation site (17%). The most common causative agents are *Staphylococcus species* (48%) [5]. The most likely cause of local SCS infections is the inoculation of implant or impulse generator pockets with microorganisms from the patient's skin flora or circulating organisms [2]. Management of infection post-SCS implantation is dependent on whether the infection is superficial or deep. In the majority of cases of deep infection, explantation of the device followed by intravenous antibiotics is necessitated [6]. The presence of a foreign body allows bacteria to form biofilms, which are critical for infection persistence. The biofilm protects the embedded bacterium from host defense mechanisms and antibiotics [7].

Infections at the superficial level (incision site) may be treated with an oral antibiotic that targets common pathogens (*staphylococci* and *streptococci*) responsible for these infections. With 7–10 days of antibiotic therapy without implant removal, superficial SSI related to SCS implants can be managed [8]. Deep infections involving the implant and/or associated complications indicate device removal. Following device removal, 7–10 days of antibiotic therapy is usually adequate for uncomplicated implant infections [9]. A SCS infection may present at different times and in different ways depending on the virulence of the causative organism and the host's immune status [5].

A study found that patients who underwent explantation had higher baseline costs, higher total costs post-implantation, and increased use of procedures to control pain. The explant cohort also demonstrated increased procedure use compared to non-explanted patients [10]. These findings suggest that patients who require device explantation may have higher healthcare resource utilization and associated costs. We report the case of a patient who developed post-incisional cellulitis and wound dehiscence over the IPG site secondary to Varicella Zoster virus (VZV) infection exactly over the incision site three-month post SCS implant.

## Case presentation

The ethics committee of our department approved the case report, and the database from Sydney Pain Management Centre was reviewed retrospectively. The patient provided written consent. The patient is an 80-year-old Lebanese male who presented to the clinic with chronic neuropathic lower back and bilateral leg pain, which was severely impacting upon his functional capacity. He had a history of long-standing lower back pain but had an exacerbation of his symptoms following two prolonged hospital admissions for (1) COVID-19 infection and (2) acute-on-chronic pain and Urinary Tract Infection (UTI). The patient had deteriorated from being functionally independent prior to hospitalization to high-care following hospitalization. Despite previously unassisted ambulation using a 4-wheel walker, the patient required assistance, displaying limited standing and terminal extension. He required two assists to sit and stand, assistance to ambulate with a four-wheeled walker and full assistance for all self-care tasks. As a result of his two hospitalizations, COVID-19 infection, acute-on-chronic pain, and UTI, his baseline health and pain levels have been adversely affected, requiring rehabilitation. Although the pain does not extend below the knees, toe numbness has developed. Leg pain surpasses back pain in intensity, with leg pain rated > 10/10 on the visual analog scale (VAS) and back pain averaging 6/10.

Physical examination was limited secondary to the patient’s functional limitations. The patient was unable to step or mobilize despite maximal support. There were no neurological deficits with 4 + /5 strength in lower extremity muscle groups and no sensory changes. His reflexes were intact. There was diffuse tenderness to palpation over all lumbar facets and paraspinal muscles. MRI of the spine revealed multilevel degenerative disc disease from L2 to S1 with grade 2 Modic changes and severe facet arthropathy at L5/S1. He had not been consulted by a neurosurgeon, though he was unlikely to be a surgical candidate based on his age and co-morbidities, which included Parkinson’s disease, coronary artery disease, osteoarthritis, osteoporosis and type II diabetes mellitus.

**Fig. 1:**
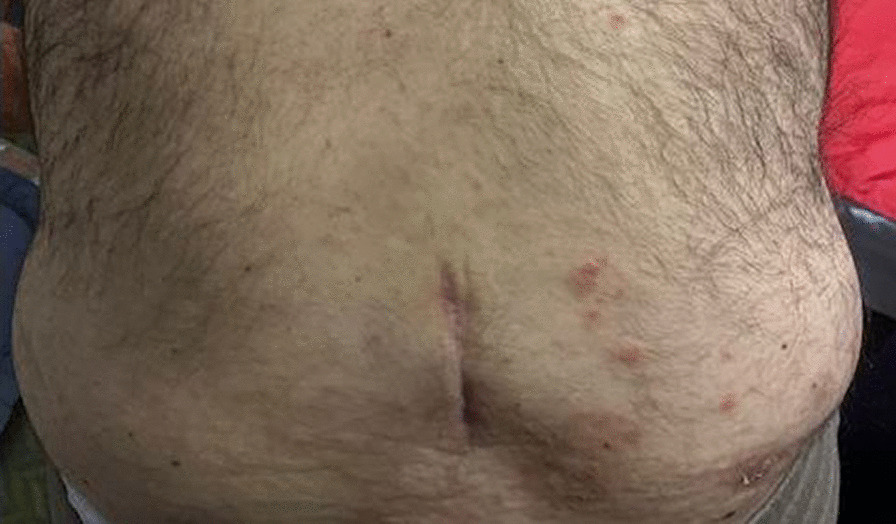
Infection in the abdominal wall after three months of SCS implantation. Shingles, also known as herpes zoster, is a painful rash caused by the varicella-zoster virus, as it can be seen in the image near the implantation. *SCS* spinal cord stimulation

**Fig. 2:**
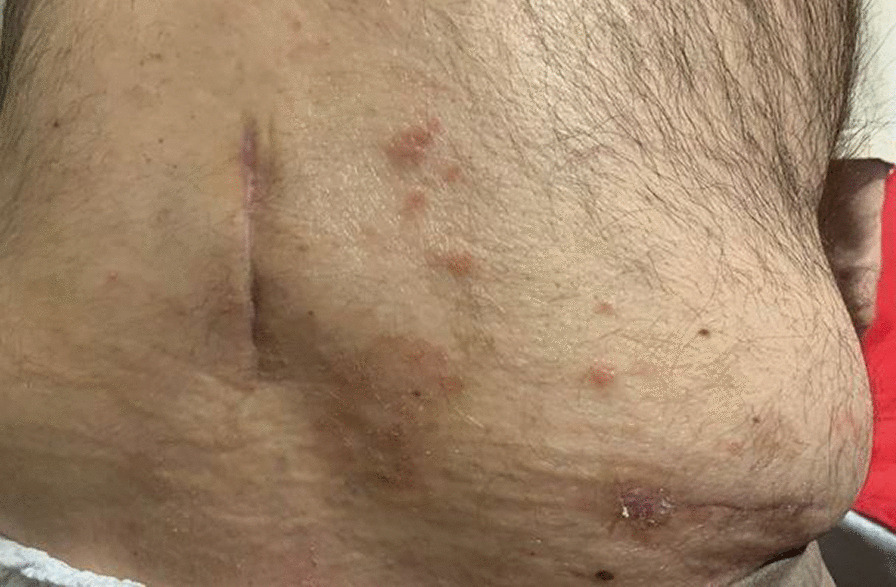
Infection in the abdominal wall after three months of SCS implantation. Shingles, also known as herpes zoster, is a painful rash caused by the varicella-zoster virus, as it can be seen in the image near the implantation. *SCS* spinal cord stimulation

**Fig. 3:**
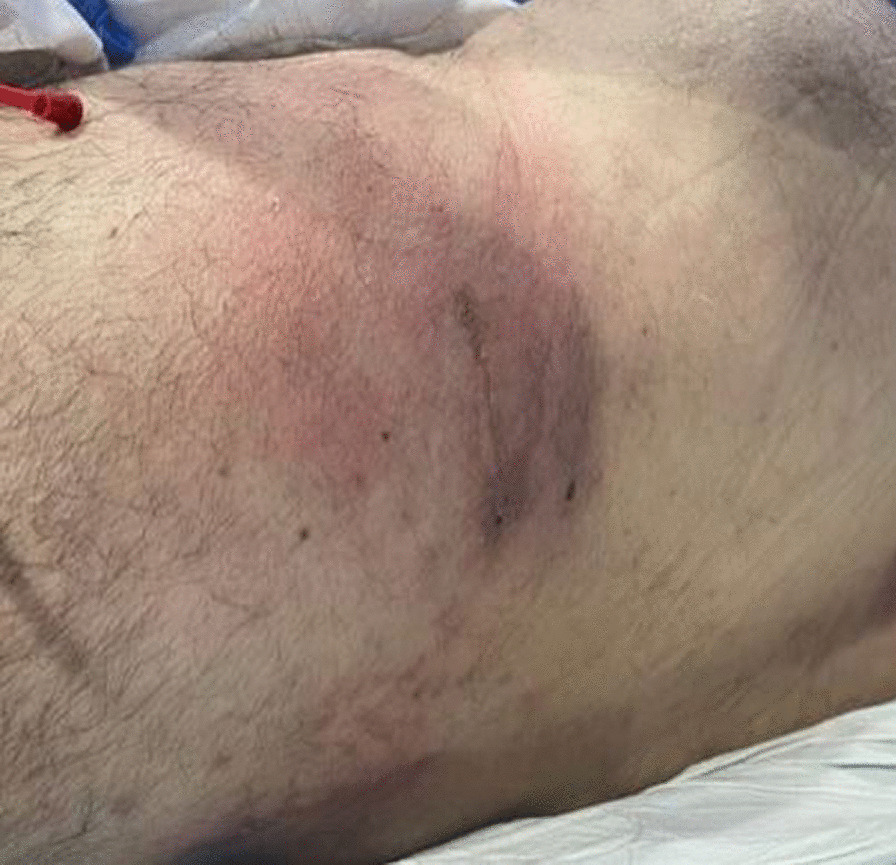
Infection in the abdominal wall after three months of SCS implantation. Shingles, also known as herpes zoster, is a painful rash caused by the varicella-zoster virus, as it can be seen in the image near the implantation. *SCS* spinal cord stimulation

The patient’s pain had been largely refractory to both conservative and interventional management. Previous lumbar facet joint injections and caudal epidural steroid injections had been largely unsuccessful, and pharmacotherapy was of limited benefit. The pain remained a significant barrier to any meaningful attempts at rehabilitation. The diagnosis involves multilevel degenerative disc disease. Treatment considerations include a potential spinal cord stimulation (SCS) trial. The trial's success parameters include at least a 50% improvement in pain severity, function, and sleep. In light of the above, the patient was deemed to be an eligible candidate for a trial of Spinal Cord Stimulation (SCS). He had a highly successful SCS trial, achieving a reduction in pain severity by 90% with subsequent improvement in physical function. He proceeded to have a permanent SCS implant four weeks later. One-month post-implant, the patient reported 0/10 pain as per the VAS and had near-complete restoration of independent functional capacity. Prior to three months post-implant, the patient developed VZV over the right flank, directly over the Implantable Pulse Generator (IPG) incision line (L2 Dermatome on the right), Figs. 1, 2, 3. His General Practitioner (GP) took a swab which was positive for VZV, and treated the superficial rash with a seven-day course of Famciclovir 250 mg TDS. Repeat swabs taken after course completion of Famciclovir were negative. Despite this, the patient developed systemic signs of infection and presented to an Emergency Department at a public tertiary hospital with a temperature of 39 °C, erythema around the IPG site in the right flank and general malaise. To determine the extent of infection, a CT scan is performed. The CT scan of the abdomen revealed no significant abnormalities except for a small fluid collection around the IPG site. The patient's laboratory results showed elevated C-reactive protein (CRP) levels of 258, WCC (White Cell Count) at 12.2 × 10^9^/L and a neutrophil count of 9.3 × 10^9^/L. In light of these findings, it was advised to aspirate the fluid under sterile conditions.

The patient was commenced on IV Flucolaxcillin, Vancomycin and a single dose of Gentamicin but showed no clinical signs of improvement. The rationale for the SCS system explantation is justified by evaluating available options [9]. While antibiotics suffice for superficial infections, deep infections necessitate removal to prevent complications. Prompt identification and intervention are crucial to minimize risks. Despite IV Flucloxacillin, Vancomycin, and Gentamicin, the patient's condition did not improve, raising a strong suspicion of a deep infection. Hence, the decision to explant the SCS system was made to effectively address the issue and prevent escalation.

Due to the patient’s deteriorating medical condition and suspected presence of deep infection around the IPG despite and a lack of growth in the culture of the fluid around the IPG, the decision to explant the SCS system was made three days post inpatient admission. Intraoperatively, a copious amount of pus was visible around the IPG. There was no evidence of pus around the lead anchor site. The patient remained stable post-operatively. Intravenous antibiotics were continued post-operatively. Swabs taken intraoperatively returned positive findings of Staphylococcus aureus colonization. The patient remained in the hospital for over one week with IV antibiotics under the guidance of Infectious Diseases specialists prior to being discharged. He made a slow recovery, given the extent of the infection, age and pre-existing medical conditions.

**Fig. 4:**
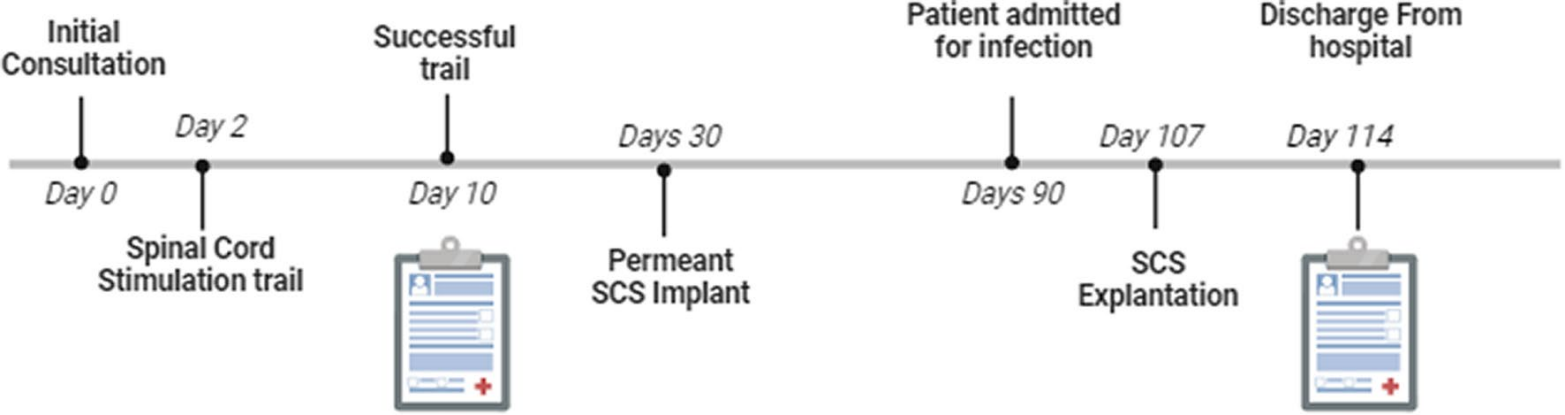
A summary of the clinical course of all follow-up visits

In the pathology tests after explantation, we analyzed Methicillin-resistant Staphylococcus aureus (MRSA) with scanty growth. Treatment with vancomycin was administered, and weekly monitoring was conducted. Two weeks after explantation, the pathology results continue to show that the patient's condition remains within normal limits, indicating a sustained resolution of the infection. Pain unfortunately returned to baseline, and he was again complaining of 8–9/10 VAS pain with limited mobility and function. As shown in Fig. 4, a summary of all follow-up visits from the initial consultation until discharge from the hospital following device explantation can be seen.

## Discussion

To the best of our knowledge, this is the first case report where spinal cord stimulator explant caused by post-incisional cellulitis secondary to Varicella Zoster Virus (shingles) Infection three months post-implant.

Secondary Varicella Zoster Virus (VZV) infection, or shingles, can contribute to bacterial infection through several mechanisms. Firstly, VZV infection manifests itself as vesicular pruritic rashes on the head, face, and trunk. These rashes can lead to breaks in the skin barrier, providing an entry point for bacteria. Bacterial superinfection of the skin is a common complication of VZV infection, which can result in bacterial colonization and subsequent infection. This is particularly relevant for elderly individuals, as they are more susceptible to shingles and are at higher risk of bacterial superinfection [11]. Furthermore, wound dehiscence, which refers to separating surgical wound edges, can also contribute to bacterial infections. Wound dehiscence is a known risk factor for surgical site infections. When a wound develops, the underlying tissues are exposed, creating an environment conducive to bacterial colonization and infection. Local wound infection, obesity, glucocorticosteroids, hypoalbuminemia, anemia, and emergency operations are additional risk factors for wound dehiscence and surgical site infections [12].

In the case of herpes zoster or shingles, it is important to note that this viral infection can lead to secondary bacterial infections. Herpes zoster is caused by the reactivation of the varicella-zoster virus (VZV) after a period of latency following primary infection with varicella (chickenpox) [13]. The reactivated virus travels from the dorsal root ganglion down the sensory nerves to the skin, causing a characteristic rash and associated symptoms [14]. The rash and associated inflammation can create an environment conducive to bacterial growth and infection, leading to post-incisional cellulitis [15]. Therefore, post-incisional cellulitis can potentially cause bacterial infection following surgery. In the case of herpes zoster or shingles, the reactivated virus can create an environment conducive to bacterial growth and infection, contributing to post-incisional cellulitis. The interesting part is the occurrence of herpes zoster infection exactly over the IPG incision, resulting in post-incisional Herpes Zoster (shingles) infection leading to cellulitis and wound dehiscence. The wound dehiscence was a later finding and was only discovered before the explantation of the IPG (no image available).

We suspect that itching around the IPG site and rupture of blisters and skin tears over the incision site contributed to the inoculation of the infection into the deeper tissue. In patients with superficial infections and no systemic symptoms, oral or intravenous antibiotics may be considered. When erythema and induration extend more than 5 cm from the wound edge, temperature > 38.5 °C, heart rate > 110 beats per minute, or WBC count > 12,000/L are present, incision and drainage or explant may be necessary [16].

Biofilms are structured communities of bacteria that adhere to surfaces and form a protective matrix. They resist antimicrobial agents and persist in the body, leading to chronic infections. Biofilm removal from spinal cord stimulation implants is challenging due to the biofilm nature and the anatomical challenges of the spinal cord region [17]. In spinal cord stimulation implants, biofilms can form on the implant surface, contributing to complications and explanations [18]. The biofilm on the SCS components is unlikely to be completely removed with irrigation and debridement, and prompt explantation of such parts is nearly always necessary [19].

The results of laboratory studies and imaging examinations may provide clinicians with information regarding the extent of an SSI and the feasibility of salvage therapy, but these data do not appear to represent a gold-standard test. According to a multicenter retrospective review of 2737 implants, a raised WBC, ESR, and CRP were only present in half of the cases of an SCS-related infection, suggesting a low sensitivity of these tests to detect infection [19].

In summary, VZV infection (shingles) and wound dehiscence can contribute to bacterial infection. The vesicular rashes associated with VZV infection can provide an entry point for bacteria, leading to skin superinfection. Additionally, wound dehiscence, which can result from tissue damage caused by VZV infection, creates an environment conducive to bacterial colonization and infection. Recognizing and managing these risk factors is important to prevent bacterial infections in individuals with secondary VZV infection and those who have undergone surgical procedures.

## Conclusion

Successful SCS implantation for chronic neuropathic pain in an 80-year-old male, resulting in SCS removal due to shingles infection caused by post-incisional cellulitis and wound dehiscence. Early detection and management are crucial. This was an extremely rare incident resulting in the loss of a highly beneficial therapy for a gentleman suffering from intractable neuropathic pain. This case highlights the need for vigilance and increased monitoring of patients who report any viral infection, particularly when the infection is in proximity to a medical device, regardless of the duration of the implant. When assessing and managing SCS infections, it is crucial to use a standardized approach. While superficial infections may be treated with antibiotics alone, deep infections may require implant removal.

To prevent post-surgical implant infections, practitioners should adhere to strict aseptic techniques during the implantation procedure. This includes proper hand hygiene, sterile drapes, gloves, and instruments. The surgical site should also be thoroughly cleaned and disinfected before the procedure. Implant infections require prompt diagnosis and treatment. If an infection is suspected, practitioners should obtain appropriate cultures and perform imaging studies to confirm the diagnosis. Treatment typically involves the removal of infected hardware and antibiotic administration [4].

## Data Availability

Data for this article are available upon request. Please contact the corresponding author for inquiries.
